# Age-related white matter alterations in children with neurofibromatosis type 1: a diffusion MRI tractography study

**DOI:** 10.3389/fnins.2025.1542957

**Published:** 2025-04-09

**Authors:** Lisa Bruckert, Katherine E. Travis, Lydia T. Tam, Kristen W. Yeom, Cynthia J. Campen

**Affiliations:** ^1^Department of Neurology, Division of Child Neurology, Palo Alto, CA, United States; ^2^Department of Pediatric, Division of Developmental-Behavioral Pediatrics, Palo Alto, CA, United States; ^3^Department of Radiology, Pediatric Radiology, Stanford, CA, United States

**Keywords:** neurofibromatosis type 1, children, white matter, diffusion MRI, tractography, fractional anisotropy, mean diffusivity

## Abstract

Neurofibromatosis type 1 (NF1) is a genetic condition affecting 1 in 3,000 children, often leading to learning challenges, including deficits in attention, executive function, and working memory. While white matter pathways play a crucial role in these cognitive processes, they are not well-characterized in NF1. In this retrospective cohort study, we used diffusion MRI tractography to examine the microstructure of major white matter pathways in 20 children with NF1 (ages 1–18 years) compared to 20 age- and sex-matched controls. An automated approach was used to identify and extract mean diffusivity (MD) and fractional anisotropy (FA) of eight cerebral white matter pathways bilaterally and the anterior and posterior part of the corpus callosum. Compared to controls, children with NF1 had significantly increased MD and significantly decreased FA in multiple white matter pathways including the anterior thalamic radiation, cingulate, uncinate fasciculus, inferior fronto-occipital fasciculus, arcuate fasciculus, and corticospinal tract. Differences in MD and FA remained significant after controlling for intracranial volume. In addition, MD and FA differences between children with NF1 and controls were greater at younger than older ages. These findings have implications for understanding the etiology of the neurocognitive deficits seen in many children with NF1.

## 1 Introduction

Neurofibromatosis type 1 (NF1) is one of the most common genetic disorders affecting ~1 in 3,000 children (Cimino and Gutmann, 2018). NF1 prevalence is not affected by sex, race, or ethnicity. Clinical manifestations vary greatly in presentation and severity and affect nearly every organ system in the body. They include changes in pigmentation like café-au-lait macules, skinfold freckling, or Lisch nodules, vasculopathy, including renal artery stenosis or moyamoya syndrome, macrocephaly, and nervous system tumors like neurofibromas or gliomas and other cancers. Children with NF1 also exhibit high rates of behavioral and learning problems that significantly impact their quality of life (Hyman et al., 2005). Specifically, 30%−70% of children with NF1 experience deficits in executive function, working memory, language, and intellectual abilities more broadly (Hyman et al., 2005; Ozonoff, 1999).

NF1 is caused by the mutation of a gene on chromosome 17, which hinders the production of the protein neurofibromin, a tumor suppressor that acts on the rat sarcoma (Ras) pathway (Bennett et al., 2003). Loss of neurofibromin and subsequent increase in Ras signaling has been shown to affect neuronal cell differentiation, growth, and apoptosis relevant to normal brain development (Dasgupta and Gutmann, 2005; Shilyansky et al., 2010; Tidyman and Rauen, 2009). In line with these pathophysiological changes in neurons' cellular processes, neuroimaging studies have revealed various brain abnormalities in NF1. These include lower cortical gyrification (Violante et al., 2013), focal hyperintense lesions on T2-weighted images, the so-called “unidentified bright objects” (Billiet et al., 2014; Szudek and Friedman, 2002), and widespread volumetric abnormalities (Huijbregts et al., 2015). For example, gray and white matter volume increases have been consistently documented in children and adolescents with NF1 relative to healthy peers (Huijbregts et al., 2015; Cutting et al., 2002; Moore et al., 2000). Similarly, the volume and thickness of the corpus callosum is increased in NF1 (Moore et al., 2000; Margariti et al., 2007). The origins and significance of these brain abnormalities, however, remain unclear.

Other frequently observed abnormalities are diffuse reductions of white matter integrity (Filippi et al., 2013; Karlsgodt et al., 2012; Zamboni et al., 2007). Diffusion magnetic resonance imaging (MRI) is commonly used to assess white matter properties *in vivo* (Catani and de Schotten, 2012). By measuring the movement of water molecules throughout the brain, diffusion MRI can provide unique insights into tissue microstructure that are otherwise not detected using conventional MRI (Barnea-Goraly et al., 2005; Mukherjee et al., 2001). The most common metrics derived from diffusion MRI are mean diffusivity (MD) and fractional anisotropy (FA). MD indexes the overall magnitude of water diffusion; FA indexes the restriction of water diffusion in a particular direction. Diffusion MRI, analyzed using tractography, allows for the interrogation of white matter properties of specific white matter pathways (Catani and de Schotten, 2012; Yeatman et al., 2012). Several tractography methods have been developed to perform the reconstruction of white matter pathways within native space, thus providing increased anatomical precision compared to ROI or whole-brain analysis methods.

To date, only a few studies have examined diffusion MRI metrics in NF1. Most of these studies used a region-of interest approach and found significantly increased MD in both hyperintense lesions and in the normal-appearing brain areas in children and adults with NF1 compared to healthy peers (Alkan et al., 2005; Eastwood et al., 2001; Tam et al., 2021; Tognini et al., 2005). Other studies used a whole-brain approach and found widespread white matter alterations as indexed by significantly increased MD and significantly decreased FA in adolescents and young adults with NF1 (Karlsgodt et al., 2012; de Blank et al., 2020; Koini et al., 2017). Only one group has used diffusion MRI tractography to reconstruct and investigate a specific white matter pathway, namely the optic pathway in NF1 (de Blank et al., 2020, 2013). In their studies, de Blank and colleagues demonstrated that (i) a decrease in FA of the optic radiations was associated with visual acuity loss in children with NF1 with optic pathway gliomas (de Blank et al., 2013) and (ii) that age-related change of diffusion MRI metrics of the optic radiations was different in children with NF1 relative to peers (de Blank et al., 2020). The latter was assessed using multiple regression analysis that examined the effect of ln(age), sex, NF1 status, and the interaction term ln(age) by NF1 status on diffusion MRI metrics. The interaction term ln(age) by NF1 status reached statistical significance in this cross-sectional study indicating that diffusion metrics of the optic radiations were reduced in young children with NF1 and matured more slowly compared to children without NF1 (de Blank et al., 2020). A similar effect of age on diffusion MRI metrics in children with NF1 was shown by Tam et al. (2021). They found that FA was reduced in the corpus callosum and frontal white matter areas in children with NF1 relative to healthy peers (Tam et al., 2021). Strikingly, the differences in FA were predominantly driven by the early and middle childhood age groups but not the adolescent one. These findings suggest potential differences in the developmental trajectory of the optic radiations and other white matter areas due to NF1.

While white matter alterations were observed throughout the brain, to our knowledge no one has reported diffusion MRI metrics controlling for known differences in intracranial volume in children with NF1 relative to controls. In addition, no study has used diffusion MRI tractography to systematically evaluate microstructural properties of white matter pathways across children and adolescents with NF1. Compared to conventional region of interest methods, diffusion MRI tractography allows the identification of the entire white matter pathway. This approach reduces interrogator bias and volume averaging while overcoming the limitations of localized analyses. By applying tractography, we can precisely map the anatomical distribution of diffusion metric changes, determine which pathways are most affected, and assess whether these alterations are localized or widespread across multiple tracts. This level of specificity is essential for linking diffusion changes to cognitive and behavioral outcomes, as different tracts serve distinct functions. Furthermore, the tractography method we employed allows for a detailed assessment and visualization of diffusion metrics along the full extent of each pathway, enabling direct comparison of tract profiles between NF1 patients and controls.

In this study, we aim to describe a comprehensive set of major white matter pathways in children with NF1 across a broad age range. Typical development of specific white matter pathways is critical for normal development of cognitive functions. Thus, our findings are important for understanding how NF1 may impact white matter development and how alterations in white matter development may contribute to cognitive outcomes in NF1. We use an automated tractography method, namely automated fiber quantification (AFQ) (Yeatman et al., 2012), to segment and characterize nine white matter pathways in children with NF1 aged 1–18 years compared to age- and sex-matched controls. AFQ allows us to identify white matter pathways in children's native space and has been shown to reliably segment white matter pathways across a wide age-range of children (Bruckert et al., 2019; Travis et al., 2016). Based on previous findings, we hypothesize that (i) children with NF1 would have higher MD and lower FA across many major white matter pathways (Karlsgodt et al., 2012; Koini et al., 2017) and that (ii) the magnitude of these differences would be affected by the age, such that MD/FA differences between children with NF1 and controls would be more pronounced in younger than older children (Tam et al., 2021; de Blank et al., 2020).

## 2 Materials and methods

### 2.1 Participants

**Figure 1:**
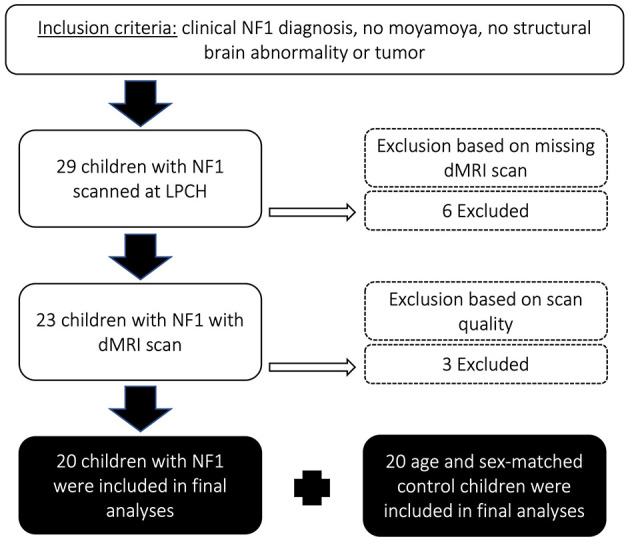
Consort flow diagram describing inclusion and exclusion criteria of pediatric patients in this study. NF1, neurofibromatosis type 1; LPCH, Lucile Packard Children's Hospital Stanford; dMRI, diffusion magnetic resonance imaging.

A database of pediatrics patients, who were treated at Lucile Packard Children's Hospital at Stanford between January 2010 and November 2017, was queried retrospectively after institutional review board approval (protocol 28674). Figure 1 depicts the consort flow diagram for inclusion and exclusion of pediatric patients. Twenty-nine children met the following inclusion criteria: (i) confirmed NF1 diagnosis, (ii) no medical history of moyamoya; (iii) no medical history of optic pathway glioma; (iv) no other intracranial mass; (v) no systemic chemotherapy. We excluded six children that were not scanned at 3T using high resolution T1-weighted volumetric and diffusion MRI. We excluded three more patients whose diffusion scans were affected by technical issues including poor whole-brain coverage (*n* = 2) and large signal dropouts (*n* = 1). Our final sample included 20 children with NF1 and 20 age- and sex-matched healthy controls (Table 1). Healthy controls were taken from the same database and included children who were scanned at the hospital for a clinical indication (e.g., isolated headaches, nausea, scalp nevus, peri-orbital dermatoid, facial hemangioma, benign strabismus without orbital or intracranial abnormality, sinus disease or inflammatory nasal obstruction, ear infection, syncope without a history of generalized seizures, and family history of aneurysm or vascular malformations) but whose brain scans were read as normal and follow-up medical assessments did not lead to a clinical diagnosis. The control cohort has been described in detail by Bruckert et al. (2019). The retrospective study involving human MRI data was approved by Stanford University Institutional Review Board (#32929). The studies were conducted in accordance with the local legislation and institutional requirements. Written informed consent for participation was not required from the participants or the participants' legal guardians/next of kin in accordance with the national legislation and institutional requirements.

**Table 1:** Characteristics of the sample.

**Group**	**Sex**	**Age (years)**	**ICV (cm^3^)**	**Relative motion**	**Clinical MRI notes/indication for clinical MRI scan**
NF1	M	1.4	1,574	0.2385	Seizures
NF1	F	2.1	1,399	0.1844	–
NF1	M	3.8	1,544	0.1397	Thickening of left optic nerve
NF1	F	3.8	1,472	0.1592	Thickening of corpus callosum
NF1	M	4.0	1,824	0.1813	Low lying cerebellar tonsils
NF1	M	6.2	2,053	0.1330	–
NF1	F	6.2	1,735	0.3432	Thickening of corpus callosum
NF1	M	7.6	1,752	0.3323	Thickening of right optic chiasm
NF1	M	8.5	1,683	0.1090	Seizures
NF1	F	9.7	1,625	0.2551	Ventriculomegaly
NF1	M	9.8	1,748	0.3148	–
NF1	M	9.8	1,933	0.1873	–
NF1	M	10.8	1,918	0.0762	–
NF1	M	13.0	1,997	0.1499	Ventriculomegaly
NF1	M	14.3	1,983	0.0893	Small arachnoid cyst
NF1	M	14.3	1,716	0.1153	–
NF1	F	14.8	1,487	0.1881	–
NF1	F	15.4	1,534	0.1262	Dysplastic clivus
NF1	F	16.8	1,378	0.2509	–
NF1	F	17.6	1,394	0.1326	–
*M* (SD) or *N*	12 Male 8 Female	9.50 (5.05)	1,687 (214.3)	0.1853 (0.0795)	
CON	M	1.2	1,247	0.2296	Headache
CON	F	2.6	1,488	0.1393	Strabismus (Brown syndrome)
CON	M	3.8	1,580	0.0799	Fevers/systemic inflammation
CON	F	3.8	1,412	0.2698	Hemangioma (left middle ear)
CON	M	4.3	1,521	0.1244	Small scalp cutis aplasia
CON	M	6.0	1,727	0.2945	Orbital lymphatic malformation
CON	F	6.4	1,486	0.2852	Headache, sinusitis
CON	M	7.9	1,690	0.2569	Sinusitis, otitis media
CON	M	8.5	1,640	0.0994	Resolved pineal cyst
CON	F	9.8	1,720	0.1358	Idiopathic optic neuritis
CON	M	9.8	1,963	0.1158	Headache
CON	M	9.8	1,598	0.1205	Nasal dermoid
CON	M	10.6	1,589	0.0818	Asymmetric pupils
CON	M	13.2	1,820	0.1489	Headache
CON	M	13.3	1,737	0.1641	Abnormal gaze
CON	M	14.4	1,482	0.1091	Headache
CON	F	15.0	1,485	0.1535	Headache
CON	F	15.8	1,670	0.1104	Headache, dizziness
CON	F	16.3	1,411	0.1358	Headache
CON	F	17.8	1,426	0.5834	Headache
*M* (SD) or *N*	12 Male 8 Female	9.52 (4.99)	1,585 (166.2)	0.1819 (0.1164)	
*p-value*		0.988	0.098^*^	0.914	

p-values < 0.1 are marked by an asterisk. Bold values shows that the *p*-values indicate significance before multiple comparison correction.

### 2.2 MRI data acquisition and preprocessing

Imaging parameters and methods for dMRI preprocessing and analyses of relative head motion have been described in previous publications (Yeatman et al., 2012; Bruckert et al., 2019; Borchers et al., 2018) and are briefly summarized below.

MRI data were obtained at 3T (GE MR750 Discovery; GE Healthcare, Waukesha, WI, USA) with an 8-channel head coil. Children between 3 months and 6 years of age were sedated under general anesthesia, and some children aged 6 years and older were sedated based on individual maturity level and ability to tolerate the MRI exam. Both high-resolution T1-weighted (3D SPGR, TR = 7.76 ms, TE = 3.47 ms, FOV = 240 × 240 mm^2^, acquisition matrix = 512 × 512, voxel size = 0.4688 × 0.4688 × 1 mm^3^, orientation = axial) and diffusion-weighted images were acquired as part of the pediatric brain MRI protocol.

High-resolution T1-weighted images were used for aligning low resolution diffusion MRI scans and to calculate the intracranial volume (ICV) for each child. FMRIB's Automated Segmentation Tool (FAST) (Zhang et al., 2001) was used to segment the T1-weighted images into three different tissue types, namely gray matter, white matter, and cerebrospinal fluid whilst also correcting for spatial intensity variations. The resulting maps of gray matter volume, white matter volume and cerebrospinal fluid volume were used to calculate ICV.

Diffusion data were collected with a twice- refocused GRAPPA DT-EPI sequence (TR = 4,000–6,000 ms depending on slice coverage, TE = 76.59 ms, FOV = 240 × 240 mm^2^, acquisition matrix = 256 × 256, voxel size = 0.9375 × 0.9375 × 3 mm^3^) using a b-value of 1,000 s/mm^2^ sampling along 25 isotropically distributed diffusion directions. One additional volume was acquired at *b* = 0 at the beginning of each scan.

We used the open-source software mrDiffusion (https://github.com/vistalab/vistasoft/tree/master/mrDiffusion) implemented in MATLAB R2014a (Mathworks, Natick, MA, United States) to preprocess the diffusion data. The non-diffusion image (b0) was registered to the subject's T1-weighted image, which had been aligned to the canonical ac-pc orientation.

Eddy current distortions and subject motion were corrected using a 14-parameter constrained non-linear co-registration approach, accounting for the expected eddy current distortions based on the phase-encoding direction of the data (Rohde et al., 2004). Each diffusion-weighted image was aligned to the b0 image using a two-stage coarse-to-fine registration maximizing normalized mutual information. The b0 image was then registered to the T1-weighted anatomical image using rigid-body transformation. The combined transform that resulted from the alignment to the T1-weighted image, eddy current correction, and motion correction was applied to the diffusion data once, and the transformed images were resampled to 2 × 2 × 2 mm^3^ isotropic voxels. Diffusion gradient directions were adjusted to fit the resampled diffusion data (Leemans and Jones, 2009). Using a standard least-squares algorithm, maps of MD and FA were generated. This preprocessing approach minimizes distortion artifacts, particularly in the frontal and temporal regions, enhancing the accuracy and reliability of diffusion measures. Relative head motion was assessed for each subject following the procedure described by Bruckert et al. (2019). No subjects were excluded due to excessive motion.

### 2.3 White matter tract identification

**Figure 2:**
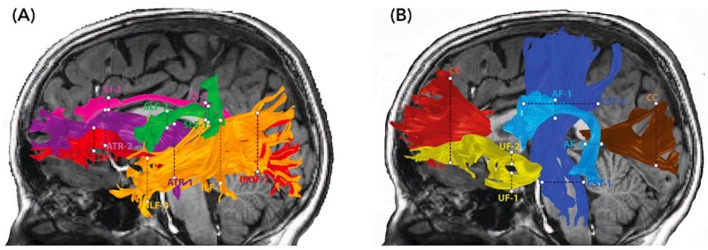
Diffusion MRI tractography of cerebral white matter pathways. Renderings of 10 major white matter pathways are overlaid on two sagittal T1-weighted images of a representative child with NF1 (age 8.5 years, male). The defining waypoint regions of interest (ROIs) are marked by dotted lines. The left panel **(A)** depicts the anterior thalamic radiation (ATR) = purple, superior longitudinal fasciculus (SLF) = green, cingulate (Ci) = pink, inferior fronto-occipital fasciculus (IFOF) = light red, and inferior longitudinal fasciculus (ILF) = orange. The right panel **(B)** depicts the corticospinal tract (CST) = dark blue, uncinate fasciculus (UF) = yellow, arcuate fasciculus (AF) = light blue, corpus callosum forceps minor (CC) = dark red, and corpus callosum forceps major (CC) = maroon.

The open-source software, Automated Fiber Quantification (AFQ) (Yeatman et al., 2012), was used to track and segment cerebral white matter pathways in each child's native space. Tractography was seeded from each voxel in a white matter mask (FA > 0.2) and deterministic tracking proceeded in all directions until FA values dropped below 0.15, or until the angle between the last path segment and next step direction was >30°. Segmentation of the bilateral anterior thalamic radiation (ATR), corticospinal tract (CST), cingulate (Cing), inferior fronto-occipital fasciculus (IFOF), inferior longitudinal fasciculus (ILF), superior longitudinal fasciculus (SLF), uncinate fasciculus (UF), arcuate fasciculus (AF), and the anterior (forceps minor, FMinor) and posterior (forceps major, FMajor) part of the corpus callosum was based on an automated waypoint ROI method implemented in AFQ (see Yeatman et al., 2012 for details). The core of the tract was calculated by defining 30 sample points along the tract and computing the robust mean position of the corresponding sample points. The robust mean was computed by estimating the three-dimensional Gaussian covariance of the sample points and removing fibers that were either more than 5 standard deviations away from the mean position of the tract or that differed more than 4 standard deviations in length from the mean length of the tract. For the SLF, we used a more rigorous cleaning approach and removed fibers that were either more than 4 standard deviations away from the mean position of the tract or that differed more than 1 standard deviations in length from the mean length of the tract. Fiber renderings for each tract and each child were visually inspected prior to any statistical analyses to ensure that each tract conformed to anatomical norms. Using these methods, we were able to identify these cerebral white matter pathways in most children. A small number of children were excluded from each analysis because a tract could not be segmented or did not conform to anatomical norms; excluded were: four (2 NF1) and six children (3 NF1) from the left and right Cing; seven (2 NF1) and two children (1 NF1) from the left and right SLF; two (0 NF1) and five children (2 NF1) from the left and right AF; two children (1 NF1) from the left ILF; one child (1 NF1) from the left IFOF; one child (0 NF1) from the left UF; and one child (0 NF1) from the FMajor. Diffusion properties (MD, FA) were quantified at 30 equidistant nodes along the central portion of each fiber tract bounded by the same two ROIs used for tract segmentation (Figure 2). Mean tract-diffusion indices were calculated by averaging MD or FA values of all 30 nodes.

### 2.4 White matter hyperintensities in patients with NF1

To assess white matter hyperintensities (WMH), an experienced neuro-oncologist and NF1 expert (CC) systematically reviewed high-resolution T1- and T2-weighted scans. Among the 20 patients with NF1, 15 had no relevant WMH (i.e., none or limited to the posterior fossa or deep gray matter nuclei), while five had WMH in the corona radiata (*N* = 2), corpus callosum (*N* = 1), and thalami (*N* = 3). Some patients had WMH in multiple regions, so the total exceeds five. Since our tractography approach reconstructs white matter pathways based on global fiber orientation rather than intensity-based segmentation, we did not explicitly exclude patient with WMH or control for WMH in our analyses. All reconstructed tracts underwent rigorous quality control to ensure anatomical accuracy.

### 2.5 Statistical analyses

Statistical analyses were conducted using IBM SPSS software (version 25.0, IBM Corporation, 2014). Statistical significance was set at *p* < 0.05. Independent *t*-tests were used to determine whether intracranial volume or relative motion during diffusion MRI differed between children with NF1 and controls. Variables that showed significant group differences were included as covariates in subsequent analyses. To assess the contribution of age to differences in white matter properties (MD and FA) between children with NF1 and controls, we conducted a series of hierarchical multiple regression analyses and included a group (NF1 vs. control) by age (in years) interaction term. If the interaction term reached statistical significance, we performed simple main effects analyses to assess the effect of age or group on MD and FA values. We used false discovery rate (FDR, *p* = 0.05) to account for multiple comparisons of the number of white matter pathways.

## 3 Results

Group characteristics of children with NF1 and controls are summarized in Table 1. By design, the groups did not differ in age or distribution of sex. The groups also did not differ in the amount of relative motion during the diffusion MRI scan. Numerically, children with NF1 had larger intracranial volume but the difference did not reach statistical significance. Because previous studies have consistently found that children with NF1 have larger brains than their healthy peers (Cutting et al., 2002; Moore et al., 2000; Karlsgodt et al., 2012; Greenwood et al., 2005) and because we saw a trend in the same direction in our cohort, intracranial volume was included as a covariate in all regression models to ensure that observed differences in MD and FA were not driven by differences in intracranial volume. Mean tract-MD and FA values for each white matter pathways for children with NF1 and their matched controls are summarized in Supplementary Table S1.

**Figure 3:**
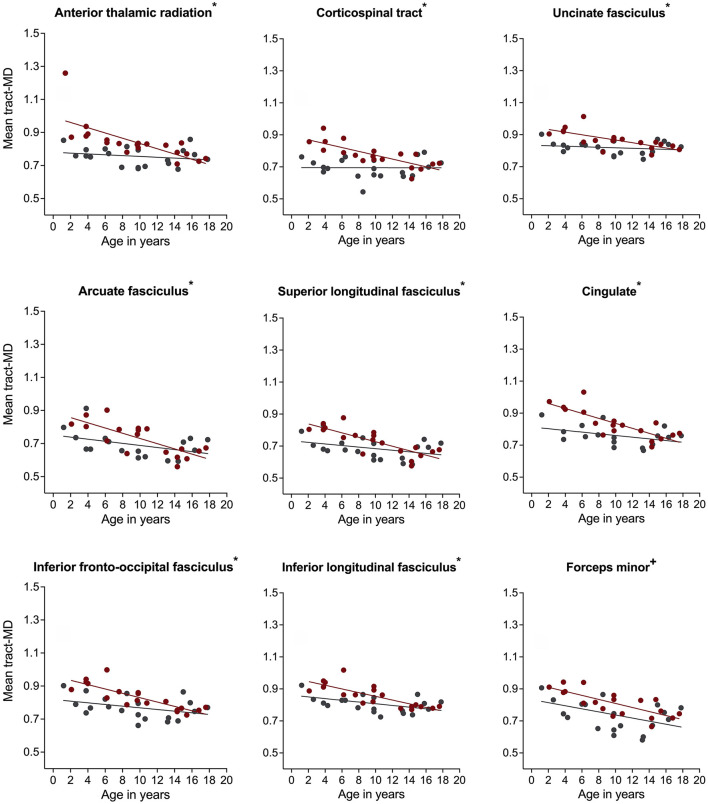
Associations of mean diffusivity (MD) with age in children with neurofibromatosis type 1 (NF1, red circles) compared to age- and sex-matched controls (CON, gray circles) after controlling for intracranial volume. Associations are shown for the right hemisphere only. Graphs marked with an *, +, and ° represent significant group-by-age interaction, significant main effect of group, and significant main effect of age respectively. Results remain significant after correcting for multiple comparisons using false discovery rate of *p* = 0.05.

For mean tract-MD, the regression analyses revealed a significant group-by-age interaction in all white matter pathways, except for the corpus callosum, FMinor, and FMajor (Table 2). Simple main effects analyses showed that children with NF1 had higher MD values compared to age- and sex-matched peers. Differences in MD were also more pronounced at younger than older ages (Figure 3 and Supplementary Figure S1). For the FMinor, and FMajor, we found a significant main effect of group. Across ages, children with NF1 had higher MD values than controls (Figure 3 and Supplementary Figure S1).

**Table 2:** Results from moderation analysis.

	**Model summary**		**Interaction: Group** ^*^ **Age**			**Main effect: Group**			**Main effect: Age**		
	**Adj**. *R*^2^	***P***	**Coeff**	**SE**	***p***	**Coeff**	**SE**	***p***	**Coeff**	**SE**	***p***
**Mean tract-MD**											
ATR-R	0.54	**<0.0001** ^*^	−0.014	0.005	**0.0046** ^*^	0.222	0.050	**0.0001**	−0.002	0.003	0.5124
ATR-L	0.52	**<0.0001** ^*^	−0.013	0.005	**0.0093** ^*^	0.208	0.053	**0.0004**	−0.004	0.004	0.2509
CST-R	0.57	**<0.0001** ^*^	−0.019	0.005	**0.0007** ^*^	0.286	0.054	< 0.0001	0.001	0.004	0.8893
CST-L	0.55	**<0.0001** ^*^	−0.015	0.005	**0.0039** ^*^	0.246	0.053	**0.0001**	−0.002	0.004	0.5646
UF-R	0.49	**0.0001** ^*^	−0.013	0.004	**0.0054** ^*^	0.197	0.048	**0.0002**	−0.001	0.003	0.6695
UF-L	0.44	**0.0003** ^*^	−0.015	0.005	**0.0068** ^*^	0.213	0.058	**0.0009**	−0.002	0.004	0.6085
Arc-R	0.48	**0.0005** ^*^	−0.010	0.005	**0.0414** ^*^	0.152	0.054	**0.0087**	−0.006	0.003	0.0740
Arc-L	0.54	**<0.0001** ^*^	−0.012	0.005	**0.0179** ^*^	0.193	0.053	**0.0009**	−0.006	0.004	0.0912
SLF-R	0.53	**<0.0001** ^*^	−0.017	0.005	**0.0044** ^*^	0.234	0.059	**0.0003**	−0.004	0.004	0.2658
SLF-L	0.56	**<0.0001** ^*^	−0.012	0.005	**0.0204** ^*^	0.191	0.053	**0.0010**	−0.007	0.004	0.0594
Cing-R	0.57	**<0.0001** ^*^	−0.10	0.004	**0.0270** ^*^	0.184	0.050	**0.0010**	−0.005	0.003	0.1118
Cing-L	0.61	**<0.0001** ^*^	−0.017	0.005	**0.0032** ^*^	0.283	0.060	< 0.0001	−0.004	0.004	0.2704
IFOF-R	0.54	**<0.0001** ^*^	−0.015	0.005	**0.0074** ^*^	0.237	0.059	**0.0003**	−0.005	0.004	0.2296
IFOF-L	0.53	**<0.0001** ^*^	−0.012	0.005	**0.0268** ^*^	0.210	0.057	**0.0008**	−0.006	0.004	0.1258
ILF-R	0.49	**0.0001** ^*^	−0.014	0.005	**0.0161** ^*^	0.207	0.059	**0.0013**	−0.005	0.004	0.2006
ILF-L	0.45	**0.0002** ^*^	−0.015	0.006	**0.0215** ^*^	0.197	0.066	**0.0051**	−0.006	0.004	0.1889
FMinor	0.52	**<0.0001** ^*^	−0.010	0.006	0.0999	0.208	0.066	**0.0035** ^*^	−0.009	0.004	0.0548
FMajor	0.37	**0.0021** ^*^	−0.017	0.008	0.0520	0.262	0.091	**0.0068** ^*^	−0.004	0.006	0.4645
**Mean tract-FA**											
ATR-R	0.65	**<0.0001** ^*^	0.008	0.002	**0.0006** ^*^	−0.141	0.023	**<0.0001**	−0.001	0.002	0.7458
ATR-L	0.62	**<0.0001** ^*^	0.010	0.002	**0.0003** ^*^	−0.141	0.026	**<0.0001**	0.000	0.002	0.8123
CST-R	0.60	**<0.0001** ^*^	0.013	0.003	**0.0001** ^*^	−0.184	0.031	**<0.0001**	−0.002	0.002	0.4512
CST-L	0.59	**<0.0001** ^*^	0.010	0.003	**0.0021** ^*^	−0.158	0.032	**<0.0001**	0.001	0.002	0.5728
UF-R	0.37	**0.0022** ^*^	0.008	0.003	**0.0037** ^*^	−0.100	0.028	**0.0009**	−0.001	0.002	0.6404
UF-L	0.34	**0.0073** ^*^	0.009	0.003	**0.0066** ^*^	−0.090	0.033	**0.0104**	−0.001	0.002	0.7949
Arc-R	0.57	**0.0002** ^*^	0.009	0.003	**0.0101** ^*^	−0.134	0.038	**0.0015**	0.001	0.002	0.5807
Arc-L	0.57	**<0.0001** ^*^	0.007	0.003	**0.0332**	−0.115	0.034	**0.0018** ^*^	0.004	0.002	0.1355
SLF-R	0.65	**<0.0001** ^*^	0.008	0.003	**0.0091** ^*^	−0.112	0.031	**0.0009**	0.006	0.002	**0.0111** ^*^
SLF-L	0.50	**0.0003** ^*^	0.001	0.003	0.7205	−0.064	0.033	0.0581	0.006	0.002	**0.0171**
Cing-R	0.52	**0.0004** ^*^	0.005	0.003	0.1328	−0.087	0.040	**0.0369** ^*^	0.005	0.003	0.0527
Cing-L	0.59	**<0.0001** ^*^	0.007	0.004	0.0914	−0.117	0.042	**0.0095** ^*^	0.006	0.003	0.0330
IFOF-R	0.65	**<0.0001** ^*^	0.004	0.003	0.1850	−0.088	0.030	**0.0066** ^*^	0.008	0.002	**0.0006** ^*^
IFOF-L	0.57	**<0.0001** ^*^	0.004	0.003	0.1773	−0.107	0.031	**0.0017** ^*^	0.003	0.002	0.1717
ILF-R	0.45	**0.0004** ^*^	0.002	0.003	0.5578	−0.045	0.028	0.1155	0.005	0.002	**0.0131** ^*^
ILF-L	0.48	**0.0002** ^*^	0.003	0.003	0.2651	−0.047	0.030	0.1199	0.005	0.002	**0.0125** ^*^
FMinor	0.62	**<0.0001** ^*^	0.006	0.003	0.0783	−0.139	0.033	**0.0002** ^*^	0.004	0.002	0.0889
FMajor	0.44	0.0005^*^	0.001	0.004	0.7285	−0.058	0.044	0.1964	0.008	0.003	0.0129^*^

FA, fractional anisotropy; MD, mean diffusivity; ATR, anterior thalamic radiation; CST, corticospinal tract; Cing, cingulum cingulate; FMajor, forceps major; FMinor, forceps minor; IFOF, inferior fronto-occipital fasciculus; ILF, inferior longitudinal fasciculus; SLF, superior longitudinal fasciculus; UF, uncinate fasciculus; Arc, arcuate fasciculus; L, left; R, right.

P-values marked with an asterisk remain significant after correcting for multiple comparisons of the number of white matter pathways using a false discovery rate of p = 0.05.

Bold p-values indicate significance before multiple comparison correction.

**Figure 4:**
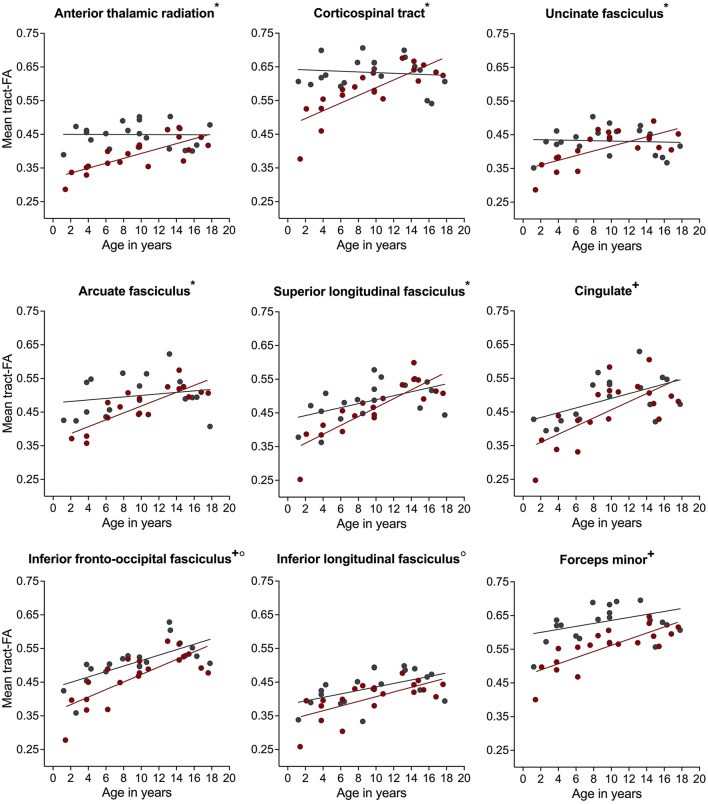
Associations of fractional anisotropy (FA) with age in children with neurofibromatosis type 1 (NF1, red circles) compared to age- and sex-matched controls (CON, gray circles) after controlling for intracranial volume. Associations are shown for the right hemisphere only. Graphs marked with an *, +, and ° represent significant group-by-age interaction, significant main effect of group, and significant main effect of age respectively. Results remain significant after correcting for multiple comparisons using false discovery rate of *p* = 0.05.

For mean tract-FA, analysis revealed a similar pattern of results. We found a significant group-by-age interaction for most white matter pathways including the bilateral ATR, CST, UF, AF, and right SLF (Table 2). Simple main effects analyses showed that children with NF1 had lower FA values compared to controls. Again, these differences were more pronounced at younger than older ages (Figure 4 and Supplementary Figure S2). For the bilateral Cing, IFOF, and FMinor, we found a significant main effect of group with lower FA values in children with NF1 relative to controls across all ages (Figure 4 and Supplementary Figure S2). For the remaining white matter pathways, namely the bilateral ILF and FMajor, we found a significant main effect of age. Across both groups, FA increased with age. All results remained significant after correcting for the number of white matter pathways using FDR.

**Figure 5:**
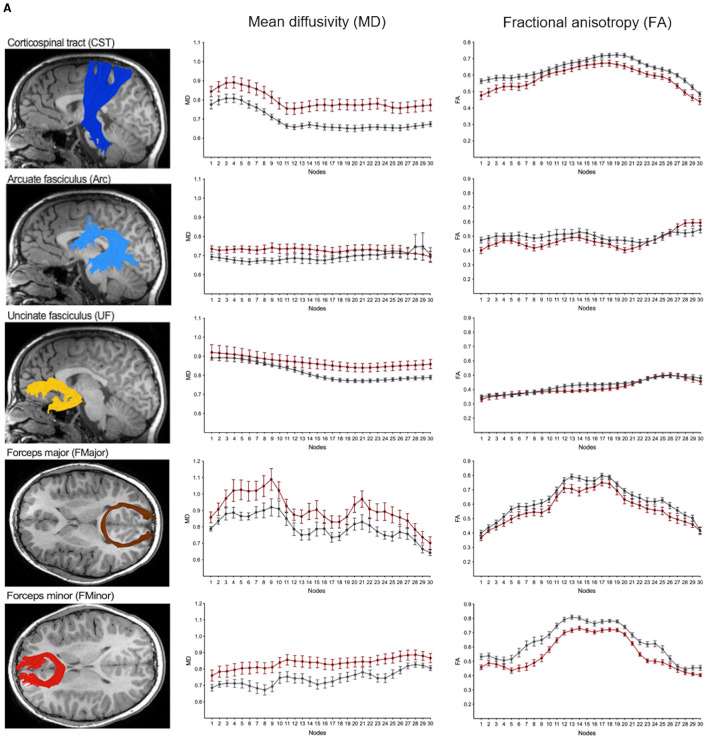
**(A)** Along-tract mean diffusivity (MD) and fractional anisotropy (FA) measures in children with neurofibromatosis type 1 (NF1, red) and age- and sex-matched controls (CON, gray). Data are shown for the right hemisphere pathways only including the anterior thalamic radiation (ATR), cingulate (Cing), inferior-fronto-occipital fasciculus (IFOF), inferior longitudinal fasciculus (ILF) and superior longitudinal fasciculus (SLF). MD and FA values sampled at 30 equidistant nodes along each white matter pathway. A corresponding reconstructed pathway from a representative patient with NF1 is overlaid on a high-resolution structural T1-weighted scan.

**Figure 6:**
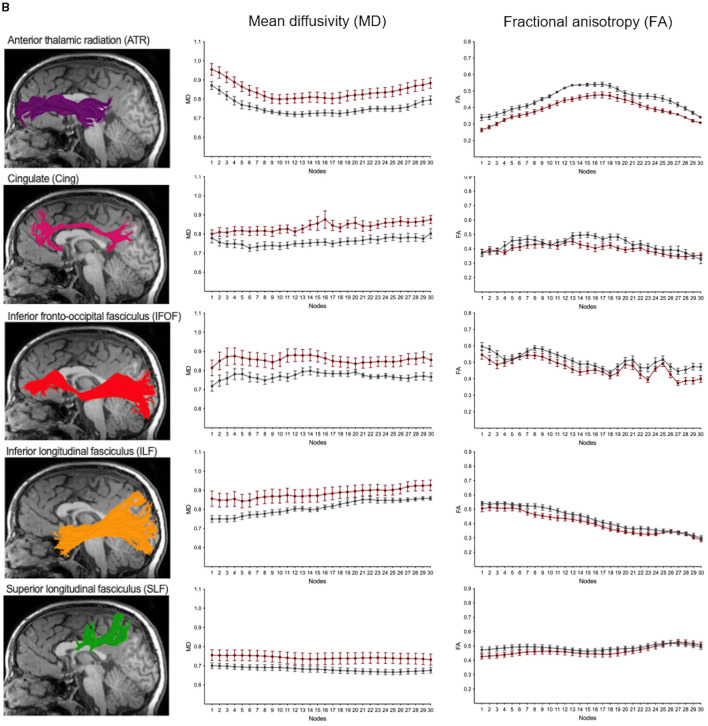
**(B)** Along-tract mean diffusivity (MD) and fractional anisotropy (FA) measures in children with neurofibromatosis type 1 (NF1, red) and age- and sex-matched controls (CON, gray). Data are shown for the right hemisphere pathways including the corticospinal tract (CST), arcuate fasciculus (AF), uncinate fasciculus (UF) as well as the commissural fibers of the forceps minor (FMinor) and forceps major (FMajor). MD and FA values sampled at 30 equidistant nodes along each white matter pathway. A corresponding reconstructed pathway from a representative patient with NF1 is overlaid on a high-resolution structural T1-weighted scan.

We performed along-tract analyses to quantify diffusion metrics at 30 equidistant nodes along each white matter pathway, generating detailed tract profiles of FA and MD. These analyses revealed widespread differences, with higher MD and lower FA in children with NF1 compared to controls across most pathways (Figures 5, 6; Supplementary Figures S3A, B). Differences were more pronounced and consistent for MD than FA values. Given our modest sample size, we did not assess statistical significance at individual nodes, as multiple comparison correction across 30 nodes for eight bilateral white matter pathways, as well as the anterior and posterior part of the corpus callosum, would not be statistically meaningful. Despite these group differences in diffusion metrics, the overall shape and trajectory of the tract profiles remained comparable between patients with NF1 and controls.

Fractional anisotropy (FA), derived from the eigenvalues of the diffusion tensor, is influenced by radial diffusivity (RD), which measures diffusivity perpendicular to the principal diffusion direction, and axial diffusivity (AD), which reflects diffusivity along the principal direction. To explore the underlying contributors to our FA findings, we also analyzed RD and AD. Regression analyses of white matter pathways with a significant FA-age interaction revealed that variations in RD, rather than AD, primarily accounted for the observed FA associations with age and group differences (Supplementary Figures S4, S5). This suggests that aberrant myelination may underlie these effects (Snook et al., 2005; Ashtari et al., 2007; Giorgio et al., 2010).

## 4 Discussion

Children with NF1 showed differences in microstructural properties of several major white matter pathways compared to age- and sex-matched controls. These differences were widespread and consistently observed in 14 out of 18 white matter pathways as quantified using diffusion MRI tractography. Moreover, these differences persisted when we controlled for intracranial volume. It is important to consider intracranial volume in studies of children with NF1 because individuals with NF1 have larger brains on average (Cutting et al., 2002; Moore et al., 2000; Karlsgodt et al., 2012; Greenwood et al., 2005).

Our results support previous studies that demonstrated increased MD and decreased FA in children and adults with NF1 compared to controls using whole-brain (Karlsgodt et al., 2012; de Blank et al., 2020) or ROI analysis (Zamboni et al., 2007; Alkan et al., 2005; Eastwood et al., 2001; Tognini et al., 2005). Karlsgodt et al. (2012), for example, found diffuse white matter differences in young adults with NF1 compared to demographically matched controls using a whole-brain analytical approach called tract-based spatial statistics. They also found significantly increased overall gray and white matter volume in the same NF1 patients but did not take these differences into account for their diffusion MRI analysis. To our knowledge this is the first study that demonstrates white matter alterations in a comprehensive set of white matter pathways controlling for overall intracranial volume.

The use of diffusion MRI tractography to examine a white matter pathway in its entirety is another novelty of this study. Only one other study used diffusion MRI tractography in patients with NF1 (de Blank et al., 2013). In their study, de Blank et al. (2013) applied tractography to assess microstructural properties of the optic pathway in children with NF1 with optic pathway gliomas. They found that a decrease in FA of the optic radiations was associated with abnormal visual acuity in children with NF1 with optic pathway gliomas. However, they did not examine other cerebral white matter pathways, assess children without glioma, or include a healthy control group. The use of tractography is advantageous, in that it can be used to reconstruct white matter pathways in native space, without registration to a template. While image registration and normalization methods have been shown to be accurate and reliable in healthy average-sized brains (Crivello et al., 2002), they can introduce biases when applied to patient populations with brain abnormalities such as gliomas or varying brain sizes across development (Brett et al., 2001; Crinion et al., 2007). Therefore, we chose tractography as the preferred method to assess white matter properties in our NF1 cohort.

Another advantage of tractography is that we obtain a detailed description of entire pathways offering a more comprehensive view of white matter alterations than traditional region-of-interest or voxel-based approaches. This level of detail is particularly valuable for studying brain-function relationships, as it enables the reconstruction and assessment of pathways implicated in specific cognitive functions. The automated tractography approach allowed for a fine-grained characterization of white matter differences across multiple pathways in NF1. These insights not only enhance our understanding of NF1-related white matter alterations but also lay essential groundwork for future research investigating longitudinal white matter changes, correlations with cognitive outcomes, and biophysical modeling in NF1. Our along-tract analyses revealed that differences in diffusion metrics extend along the entire length of the affected pathways, with higher MD and lower FA in children with NF1 compared to controls. Importantly, the overall shape and trajectory of the tract profiles remained comparable between groups, and the FA tract profiles closely resembled those reported by Yeatman et al. (2012), suggesting that tract reconstruction was anatomically consistent across participants. This supports the robustness of our tractography approach and indicates that the observed group differences are unlikely to be driven by segmentation errors. Moreover, the along-tract analyses suggest that the differences identified in our main analysis are not confined to specific focal regions but instead extend along the entirety of the white matter pathways. This widespread pattern of alterations aligns with the hypothesis that NF1-related microstructural changes reflect a more global disruption of white matter integrity rather than localized abnormalities.

A potential factor influencing diffusion metrics is the presence of WMH, particularly when white matter pathways pass through affected regions. However, in our relatively healthy NF1 cohort—without cases of moyamoya disease, optic pathway gliomas, intracranial masses, or systemic chemotherapy—WMH were present in only five patients. These WMH were located in regions traversed by major white matter pathways, including the CST, ATR, SLF, corpus callosum, and cingulum. Our rigorous quality control process ensured that reconstructed tracts adhered to known anatomical trajectories, minimizing the likelihood that WMH significantly influenced tractography results. Furthermore, our along-tract diffusion analysis demonstrated that group differences in FA and MD were distributed along the entire length of these pathways rather than being confined to specific focal locations, suggesting that WMH did not systematically drive our findings. Nonetheless, future studies with larger cohorts and advanced imaging techniques, such as myelin-sensitive MRI, are needed to further investigate the relationship between WMH and white matter microstructure in NF1.

We corroborated findings from our group (Tam et al., 2021) and other researchers (de Blank et al., 2020) that the magnitude of white matter differences between children with NF1 and controls varies by age. Prior reports were limited to ROI analyses of the fronto-temporal white matter, whole-brain analysis, and tractography of the optic pathway only. The current work characterizes a comprehensive set of nine major white matter pathways bilaterally and the corpus callosum throughout the pediatric NF1 brain. Our results indicate that in most white matter pathways, differences were larger at younger ages. While this study did not assess individual children longitudinally, our findings suggest that children with NF1 may have altered or delayed white matter maturation that can lead to persistent white matter abnormalities later in life. Patients with NF1 have previously been shown to have abnormal diffusion metrics (Tam et al., 2021; de Blank et al., 2020). Most diffusion MRI studies, however, have focused on adult patients with NF1 (Karlsgodt et al., 2012; Zamboni et al., 2007) limiting our understanding of NF1-related white matter differences in the developing brain. The definition of normative values for diffusion MRI metrics across ages is an essential step in developing potential diffusion MRI biomarkers, which in turn could facilitate early identification of children with NF1 at risk for neurocognitive or visual deficits. Diffusion MRI studies that have examined children with NF1 have either not assessed the effect of age on diffusion metrics (Filippi et al., 2013) or focused on whole-brain analysis with a single white matter pathway (Karlsgodt et al., 2012; de Blank et al., 2020) or were restricted to a set of ROIs (Tam et al., 2021).

Results from these and our cross-sectional diffusion MRI study in children with NF1 suggest that white matter differences may not be static throughout a patient's life but that they change due to maturation and experience-dependent reorganization. Most work on white matter plasticity has focused on myelination, the process of coating axons with a fatty myelin sheath to improve signal conduction (Freeman et al., 2016; Hartline and Colman, 2007). Consistent with postmortem studies (Benes et al., 1994), neuroimaging studies have shown that the most dynamic period of myelination occurs in the first few years of life coinciding with the rapid increase in cognitive abilities (Dean et al., 2015; Deoni et al., 2012). White matter maturation, however, continues well into adolescence and adulthood (Lebel and Beaulieu, 2011; Sexton et al., 2014). Longitudinal studies in children with NF1 are needed to learn if and how NF1-related white matter alterations change over time and to determine if there is a specific window of opportunity during childhood for targeted treatment to delay or moderate any white matter alterations. In addition, future neuroimaging studies should combine diffusion MRI with imaging methods that can provide direct measures of myelin content (Mezer et al., 2013) to triangulate the underlying biological mechanisms.

The data in this study were analyzed retrospectively and therefore we were limited in our sample size. A sample size of 20 children with NF1 and 20 well-matched controls, however, is comparable (or higher) to previous diffusion MRI studies in NF1 (Filippi et al., 2013; Karlsgodt et al., 2012; Koini et al., 2017; de Blank et al., 2013). The retrospective design of this study also limited the available imaging data as neither longitudinal scans nor additional modalities, such as quantitative T1 mapping, were included in the pediatric MRI brain protocol. Because our diffusion MRI data were clinically acquired with routine parameters (25 diffusion directions, a single b0 image, and a *b*-value of 1,000 s/mm^2^), the use of advanced diffusion modeling techniques and biophysical white matter models was inherently restricted. DTI metrics, such as FA and MD, are highly sensitive to microstructural differences, making them valuable for detecting white matter alterations. However, their specificity is limited, as they cannot distinguish between underlying biological processes such as changes in axonal density, myelination, or extracellular water content. Future studies should address these limitations by incorporating (1) longitudinal data to track white matter changes over time; (2) imaging methods that directly measure myelin content; and (3) advanced diffusion MRI acquisition parameters that support biophysical models like NODDI, which can provide more specific insights into white matter microstructure by disentangling these distinct contributions. Longitudinal MRI studies in NF1 are particularly crucial for identifying specific periods of altered white matter maturation and determining whether there is a developmental window in which targeted interventions could mitigate or delay these changes. Repeated diffusion and myelin-sensitive imaging at different developmental stages could provide critical insights into the trajectory of white matter alterations in NF1, their potential impact on cognitive and functional outcomes, and opportunities for early intervention. Given the potential clinical implications, these research directions remain an urgent priority.

Further, Lucile Packard Children's Hospital at Stanford is a tertiary-care pediatric hospital, and our patients may harbor more severe disease than the average child with NF1. However, we excluded children with NF1 with moyamoya, glioma, or who had received chemotherapy to limit our sample to children without visible white matter injury or other significant brain abnormalities. In this study, we capitalized on the availability of scans acquired in children who presented at our hospital for a clinical indication as our control cohort. This may make the scans unrepresentative of a community-based sample, however, our thorough chart review (for details see Bruckert et al., 2019) combined with our strict inclusion and exclusion criteria make this possibility unlikely. MRI scans and follow-up reports of all our control participants were normal as reviewed by a pediatric neuroradiologist and developmental-behavioral pediatrician.

## 5 Conclusion

We validated prior findings that white matter microstructure is altered in the pediatric NF1 brain, and that white matter differences are more pronounced in younger children with NF1 compared to healthy peers. Using tractography our study provided increased specificity for a comprehensive set of white matter pathways. In addition, our findings suggest that diffusion MRI abnormalities are due to differences in white matter microstructure rather than differences in brain volume seen in NF1. These differences may point to aberrant oligodendroglial precursor cell dynamics during childhood and may have implication for cognitive development and glioma genesis in NF1. Children with NF1 harbor high rates of learning challenges including attention, executive function, and processing disorders and high rates of low-grade gliomas. Future studies should investigate the relationship of white matter pathways implicated in cognition or affected by gliomas and neurocognitive and clinical outcome to understand the clinical relevance of the observed white matter alterations.

## Data Availability

The raw data supporting the conclusions of this article will be made available by the authors, without undue reservation.
