# Supplementary material for: Age-related white matter alterations in children with neurofibromatosis type 1: a diffusion MRI tractography study
Source: Front Neurosci. 2025 Apr 9;19:1542957. doi: 10.3389/fnins.2025.1542957 (PMC12016576; doi:10.3389/fnins.2025.1542957)
Supplement: Supplementary file 1 [file Data_Sheet_1.pdf]

# *Supplementary Material for:*

## "Age-related White Matter Alterations in Children with Neurofibromatosis Type 1: A Diffusion MRI Tractography Study"

Bruckert, L., Travis, K. E., Tam, L. T., Yeom, K. W., and Campen, C. J.

## 1 Supplementary Figures and Tables

### 1.1 Supplementary Tables

**Table S1.** Mean(M) and standard deviation (SD) of tract-mean diffusivity (MD) and tract-fractional anisotropy (FA) of major white matter pathway in children with neurofibromatosis type 1 (NF1) and age- and sex-matched controls (CON).

| | | Tract-MD | | Tract-FA | |
|---|---|---|---|---|---|
| | N | NF1 *M (SD)* | CON *M (SD)* | NF1 *M (SD)* | CON *M (SD)* |
| ATR-R | 40 | 0.841 (0.113) | 0.756 (0.055) | 0.389 (0.047) | 0.449 (0.036) |
| ATR-L | 40 | 0.798 (0.127) | 0.727 (0.054) | 0.419 (0.063) | 0.460 (0.039) |
| CST-R | 40 | 0.797 (0.126) | 0.695 (0.060) | 0.582 (0.072) | 0.633 (0.046) |
| CST-L | 40 | 0.789 (0.126) | 0.699 (0.056) | 0.592 (0.072) | 0.646 (0.051) |
| UF-R | 40 | 0.889 (0.110) | 0.819 (0.040) | 0.412 (0.051) | 0.432 (0.041) |
| UF-L | 40 | 0.844 (0.136) | 0.783 (0.045) | 0.435 (0.067) | 0.442 (0.048) |
| Arc-R | 35 | 0.727 (0.097) | 0.693 (0.080) | 0.471 (0.059) | 0.498 (0.060) |
| Arc-L | 40 | 0.818 (0.129) | 0.743 (0.057) | 0.458 (0.070) | 0.494 (0.059) |
| SLF-R | 40 | 0.755 (0.144) | 0.686 (0.065) | 0.459 (0.078) | 0.488 (0.058) |
| SLF-L | 40 | 0.791 (0.128) | 0.718 (0.067) | 0.412 (0.055) | 0.451 (0.063) |
| Cing-R | 35 | 0.832 (0.091) | 0.759 (0.063) | 0.398 (0.063) | 0.431 (0.051) |
| Cing-L | 38 | 0.835 (0.143) | 0.732 (0.063) | 0.454 (0.089) | 0.488 (0.065) |
| IFOF-R | 40 | 0.857 (0.138) | 0.771 (0.068) | 0.467 (0.073) | 0.510 (0.058) |
| IFOF-L | 39 | 0.872 (0.133) | 0.785 (0.063) | 0.458 (0.058) | 0.513 (0.058) |
| ILF-R | 40 | 0.882 (0.140) | 0.810 (0.055) | 0.403 (0.052) | 0.433 (0.050) |
| ILF-L | 40 | 0.899 (0.156) | 0.842 (0.057) | 0.420 (0.059) | 0.436 (0.047) |
| FMinor | 40 | 0.836 (0137) | 0.742 (0.102) | 0.557 (0.059) | 0.633 (0.063) |
| FMajor | 40 | 0.907 (0.183) | 0.800 (0.097) | 0.572 (0.086) | 0.625 (0.053) |

ATR = anterior thalamic radiation, CST = corticospinal tract, Cing = cingulum cingulate, FMajor = forceps major, FMinor = forceps minor, IFOF = inferior fronto-occipital fasciculus, ILF = inferior longitudinal fasciculus, SLF = superior longitudinal fasciculus, UF = uncinate fasciculus, Arc = arcuate fasciculus, L = left / R = right

**Table S2.** Mean(M) and standard deviation (SD) of tract-radial diffusivity (RD) and tract-axial diffusivity (AD) of major white matter pathway in children with neurofibromatosis type 1 (NF1) and age- and sex-matched controls (CON).

| | | Tract-RD | | Tract-AD | |
|---|---|---|---|---|---|
| | **N** | **NF1** *M (SD)* | **CON** *M (SD)* | **NF1** *M (SD)* | **CON** *M (SD)* |
| ATR-R | 40 | 0.652 (0.113) | 0.556 (0.055) | 1.198 (0.056) | 1.157 (0.065) |
| ATR-L | 40 | 0.604 (0.131) | 0.530 (0.054) | 1.163 (0.068) | 1.121 (0.067) |
| CST-R | 40 | 0.498 (0.132) | 0.400 (0.057) | 1.375 (0.088) | 1.284 (0.088) |
| CST-L | 40 | 0.487 (0.131) | 0.393 (0.058) | 1.373 (0.098) | 1.310 (0.093) |
| UF-R | 40 | 0.672 (0.115) | 0.609 (0.050) | 1.301 (0.060) | 1.239 (0.040) |
| UF-L | 40 | 0.629 (0.144) | 0.577 (0.054) | 1.253 (0.070) | 1.195 (0.054) |
| Arc-R | 35 | 0.525 (0.095) | 0.490 (0.083) | 1.130 (0.122) | 1.097 (0.091) |
| Arc-L | 40 | 0.601 (0.138) | 0.527 (0.070) | 1.229 (0.056) | 1.173 (0.066) |
| SLF-R | 40 | 0.557 (0.154) | 0.491 (0.071) | 1.128 (0.074) | 1.077 (0.075) |
| SLF-L | 40 | 0.606 (0.126) | 0.533 (0.081) | 1.135 (0.079) | 1.086 (0.050) |
| Cing-R | 35 | 0.642 (0.102) | 0.566 (0.061) | 1.212 (0.089) | 1.146 (0.099) |
| Cing-L | 38 | 0.611 (0.161) | 0.514 (0.067) | 1.276 (0.133) | 1.197 (0.067) |
| IFOF-R | 40 | 0.620 (0.150) | 0.530 (0.070) | 1.329 (0.125) | 1.251 (0.098) |
| IFOF-L | 39 | 0.637 (0.136) | 0.540 (0.070) | 1.344 (0.142) | 1.275 (0.084) |
| ILF-R | 40 | 0.682 (0.144) | 0.607 (0.066) | 1.281 (0.139) | 1.216 (0.053) |
| ILF-L | 40 | 0.680 (0.157) | 0.627 (0.064) | 1.337 (0.165) | 1.271 (0.068) |
| FMinor | 40 | 0.538 (0.133) | 0.429 (0.093) | 1.431 (0.153) | 1.368 (0.140) |
| FMajor | 40 | 0.577 (0.186) | 0.467 (0.083) | 1.568 (0.203) | 1.465 (0.155) |

ATR = anterior thalamic radiation, CST = corticospinal tract, Cing = cingulum cingulate, FMajor = forceps major, FMinor = forceps minor, IFOF = inferior fronto-occipital fasciculus, ILF = inferior longitudinal fasciculus, SLF = superior longitudinal fasciculus, UF = uncinate fasciculus, Arc = arcuate fasciculus, L = left / R = right

## 1.2 Supplementary Figures

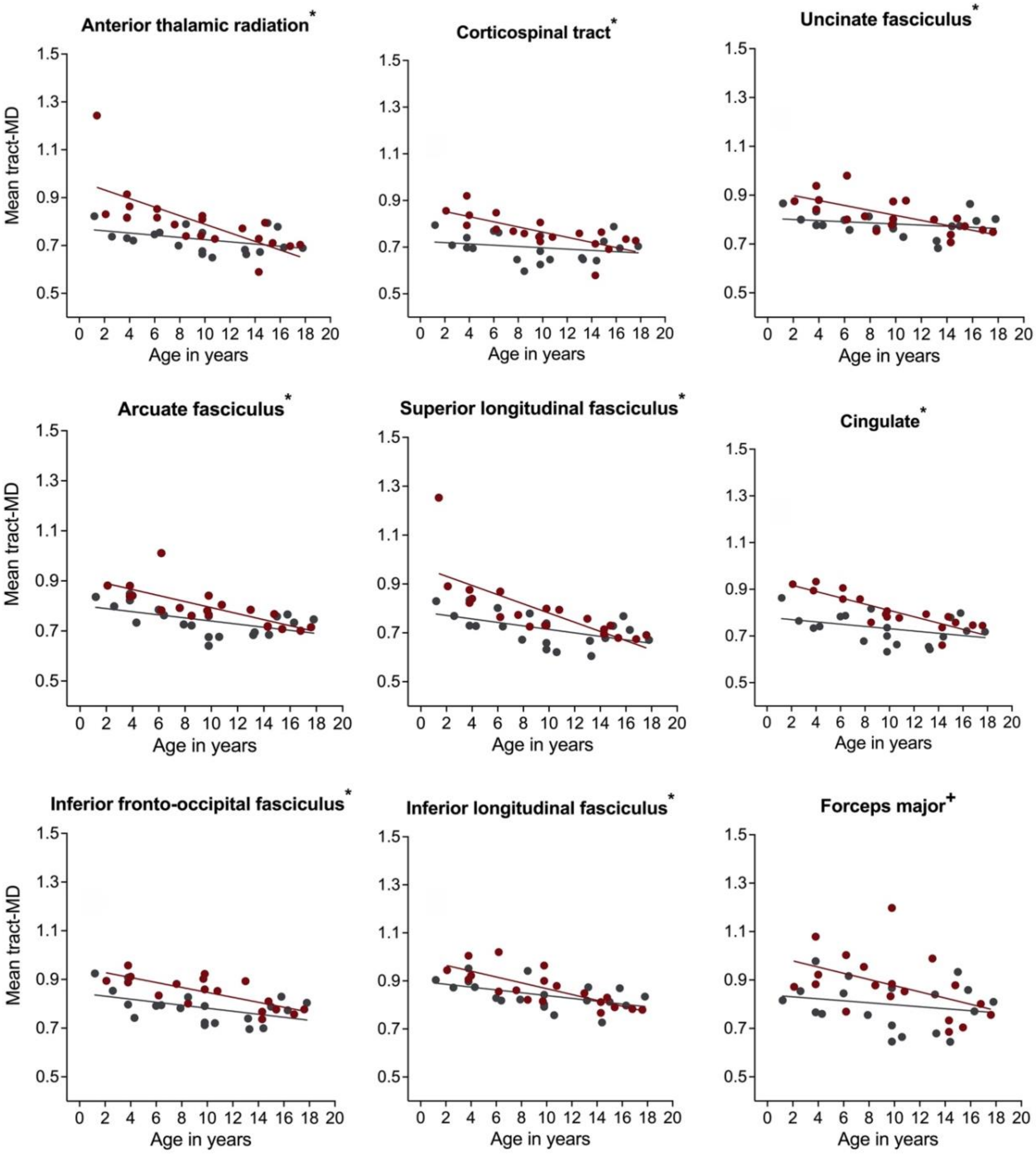


**Supplementary Figure 1. Associations of mean diffusivity (MD) with age in children with neurofibromatosis type 1 (NF1, red circles) compared to age- and sex-matched controls (CON, grey circles) after controlling for intracranial volume.** Associations are shown for the left hemisphere only. Graphs marked with an *, +, and ° represent significant group-by-age interaction, significant main effect of group, and significant main effect of age respectively. Results remain significant after correcting for multiple comparisons using false discovery rate of $p = 0.05$.

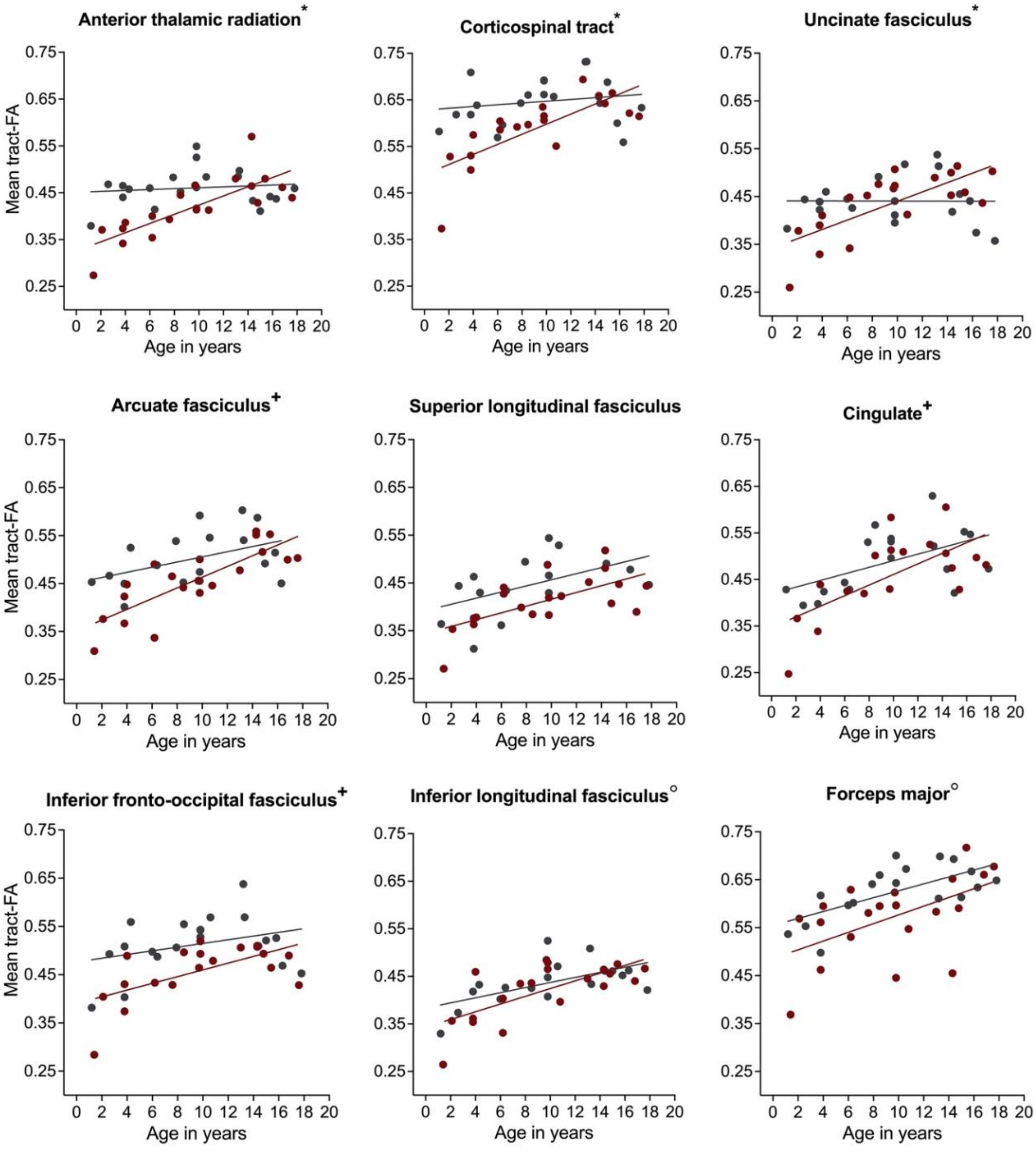


**Supplementary Figure 2. Associations of fractional anisotropy (FA) with age in children with neurofibromatosis type 1 (NF1, red circles) compared to age- and sex-matched controls (CON, grey circles) after controlling for intracranial volume.** Associations are shown for the <u>left</u> hemisphere only. Graphs marked with an *, +, and ° represent significant group-by-age interaction, significant main effect of group, and significant main effect of age respectively. Results remain significant after correcting for multiple comparisons using false discovery rate of $p = 0.05$.

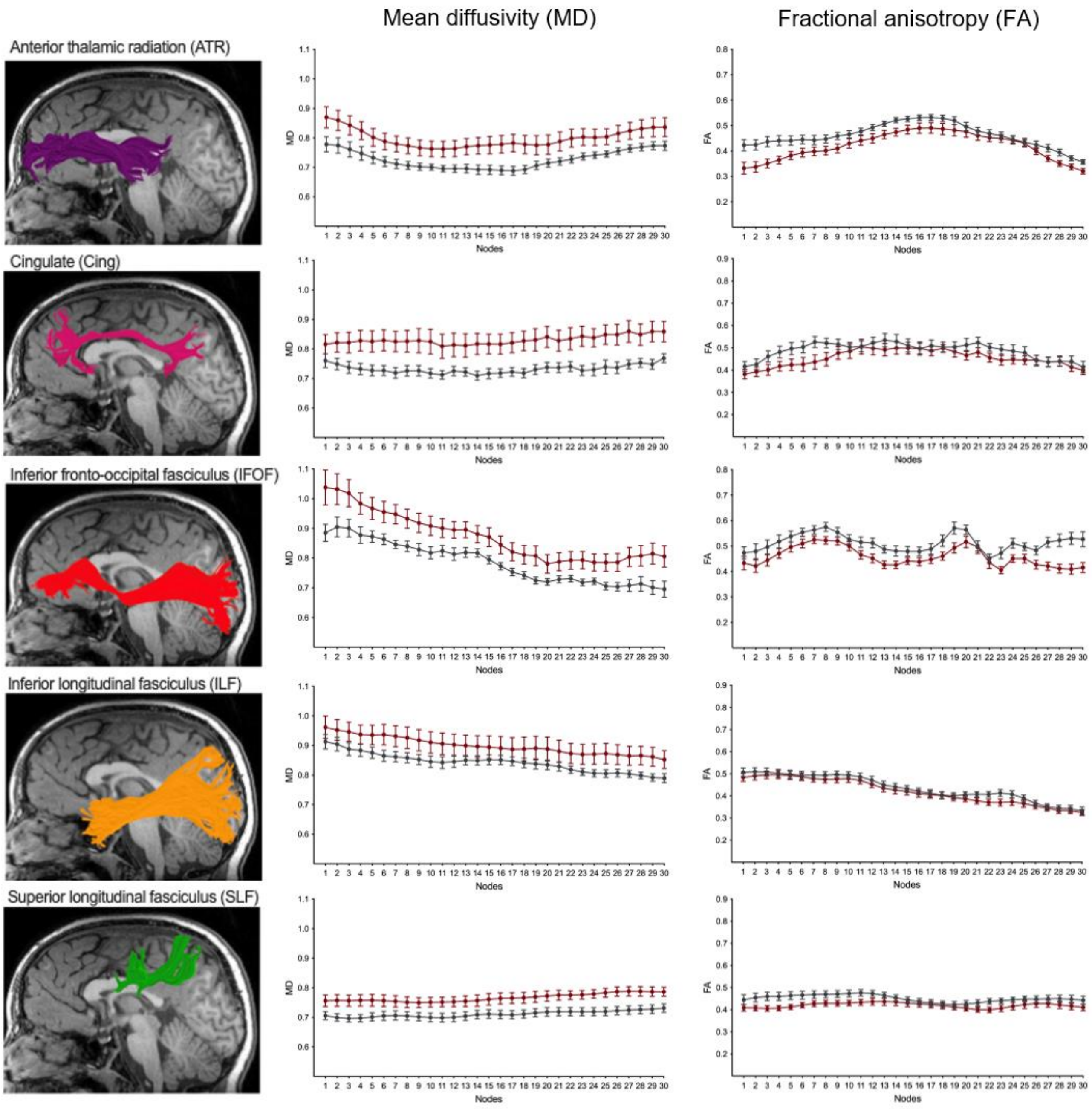


**Supplementary Figure 3A. Along-tract mean diffusivity (MD) and fractional anisotropy (FA) measures in children with neurofibromatosis type 1 (NF1, red) and age- and sex-matched controls (CON, gray).** Data are shown for the left hemisphere pathways only including the anterior thalamic radiation (ATR), cingulate (Cing), inferior-fronto-occipital fasciculus (IFOF), inferior longitudinal fasciculus (ILF) and superior longitudinal fasciculus (SLF). MD and FA values sampled at 30 equidistant nodes along each white matter pathway. A corresponding reconstructed pathway from a representative patient with NF1 is overlaid on a high-resolution structural T1-weighted scan.

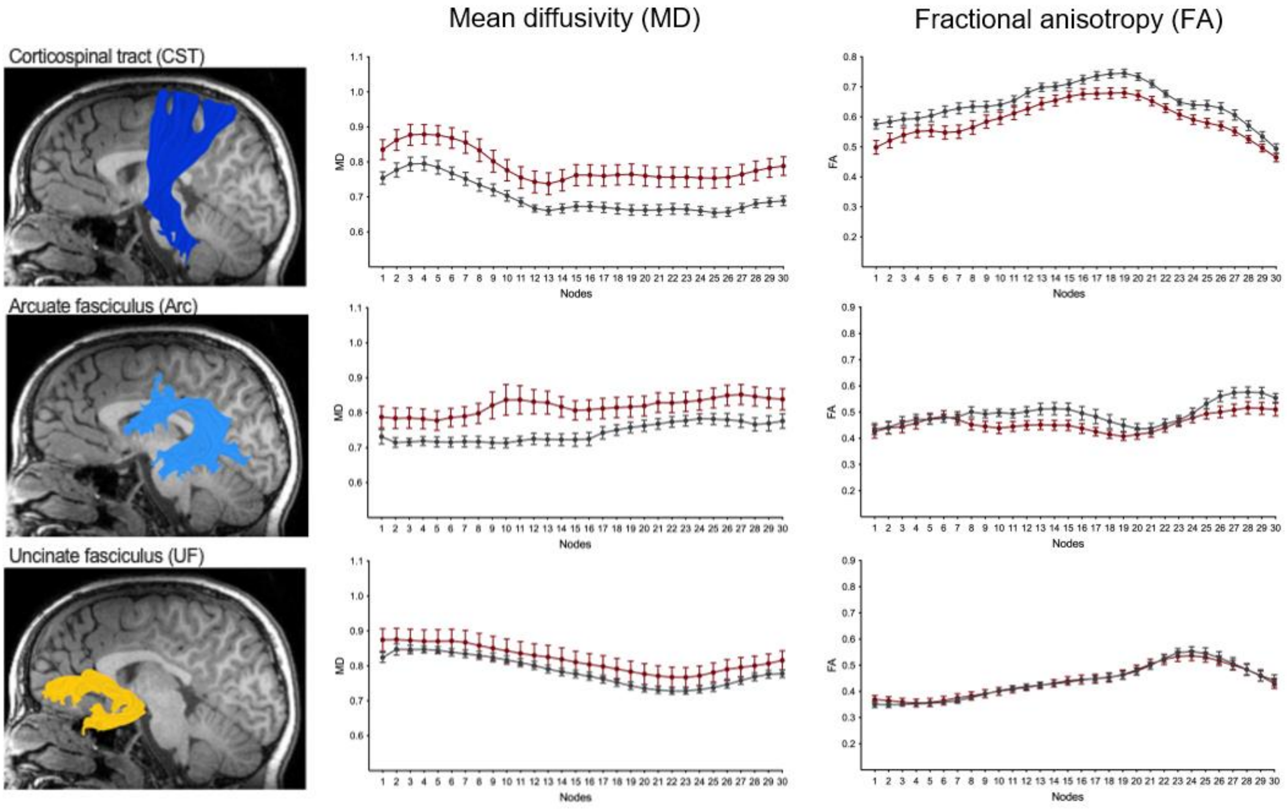


**Supplementary Figure 3B. Along-tract mean diffusivity (MD) and fractional anisotropy (FA) measures in children with neurofibromatosis type 1 (NF1, red) and age- and sex-matched controls (CON, gray).** Data are shown for the left hemisphere pathways only. MD and FA values sampled at 30 equidistant nodes along each white matter pathway. A corresponding reconstructed pathway from a representative patient with NF1 is overlaid on a high-resolution structural T1-weighted scan.

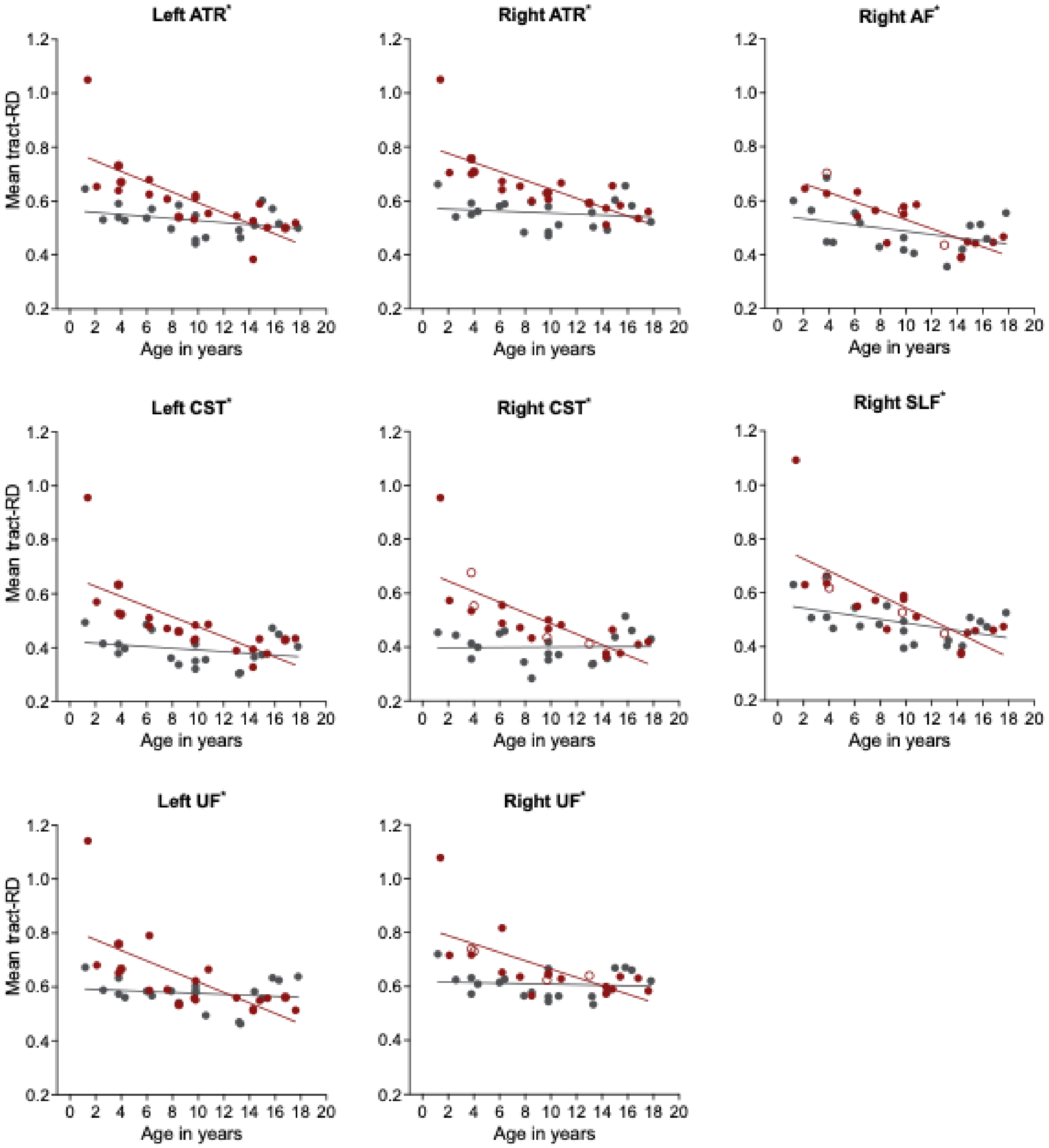


**Supplementary Figure 4. Associations of radial diffusivity (RD) with age in children with neurofibromatosis type 1 (NF1, red circles) compared to age- and sex-matched controls (CON, grey circles) after controlling for intracranial volume.** Associations are shown for white matter pathways that previously exhibited a significant FA–age interaction. Graphs marked with *, +, and ° indicate a significant group-by-age interaction, a significant main effect of group, and a significant main effect of age, respectively. All results remain significant after false discovery rate (FDR) correction at $p = 0.05$, except for the right AF.

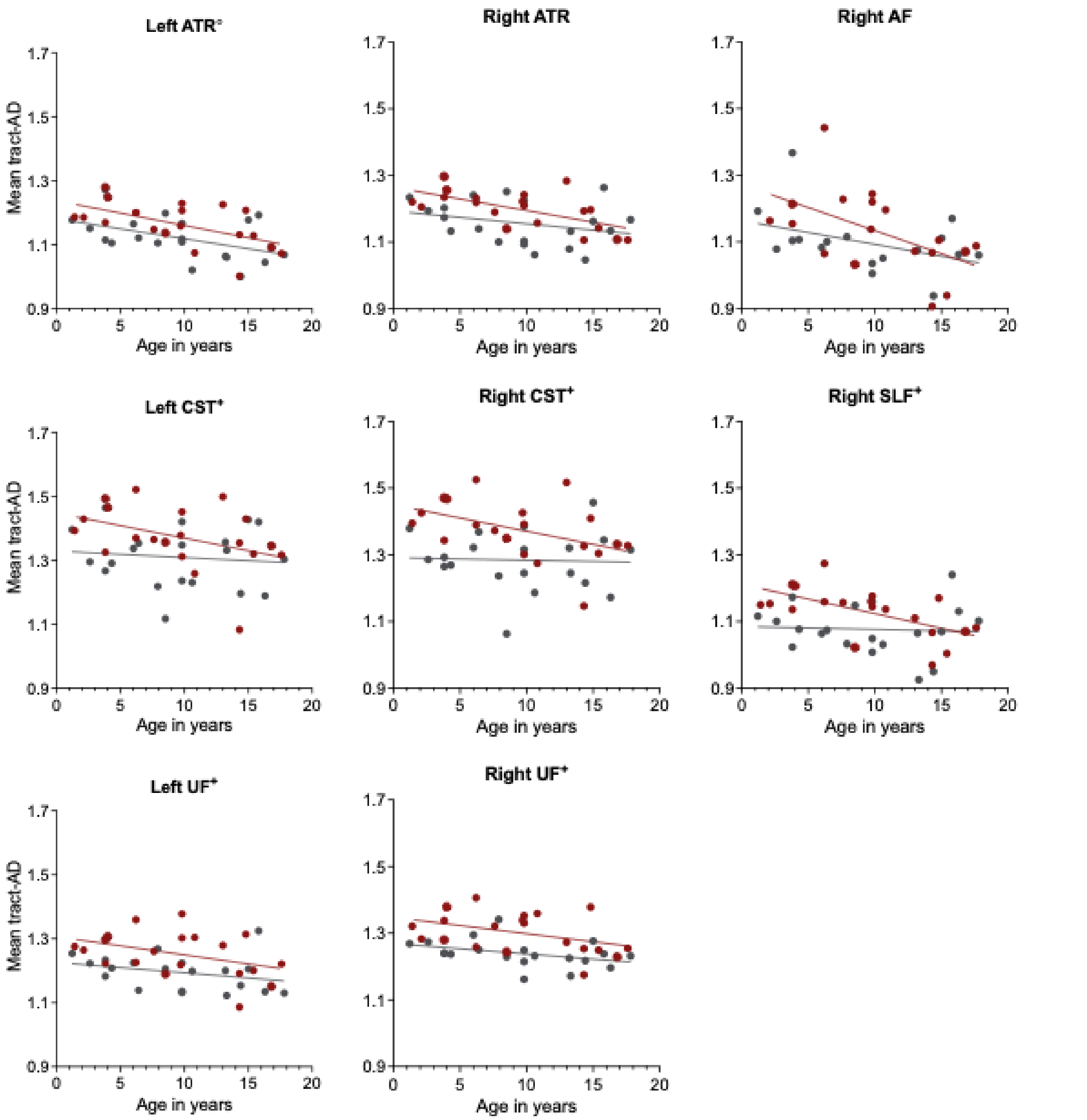


**Supplementary Figure 5. Associations of axial diffusivity (AD) with age in children with neurofibromatosis type 1 (NF1, red circles) compared to age- and sex-matched controls (CON, grey circles) after controlling for intracranial volume.** Associations are shown for white matter pathways that previously exhibited a significant FA–age interaction. Graphs marked with *, +, and ° indicate a significant group-by-age interaction, a significant main effect of group, and a significant main effect of age, respectively. Results remain significant after false discovery rate (FDR) correction at p = 0.05 for the right CST and bilateral UF.

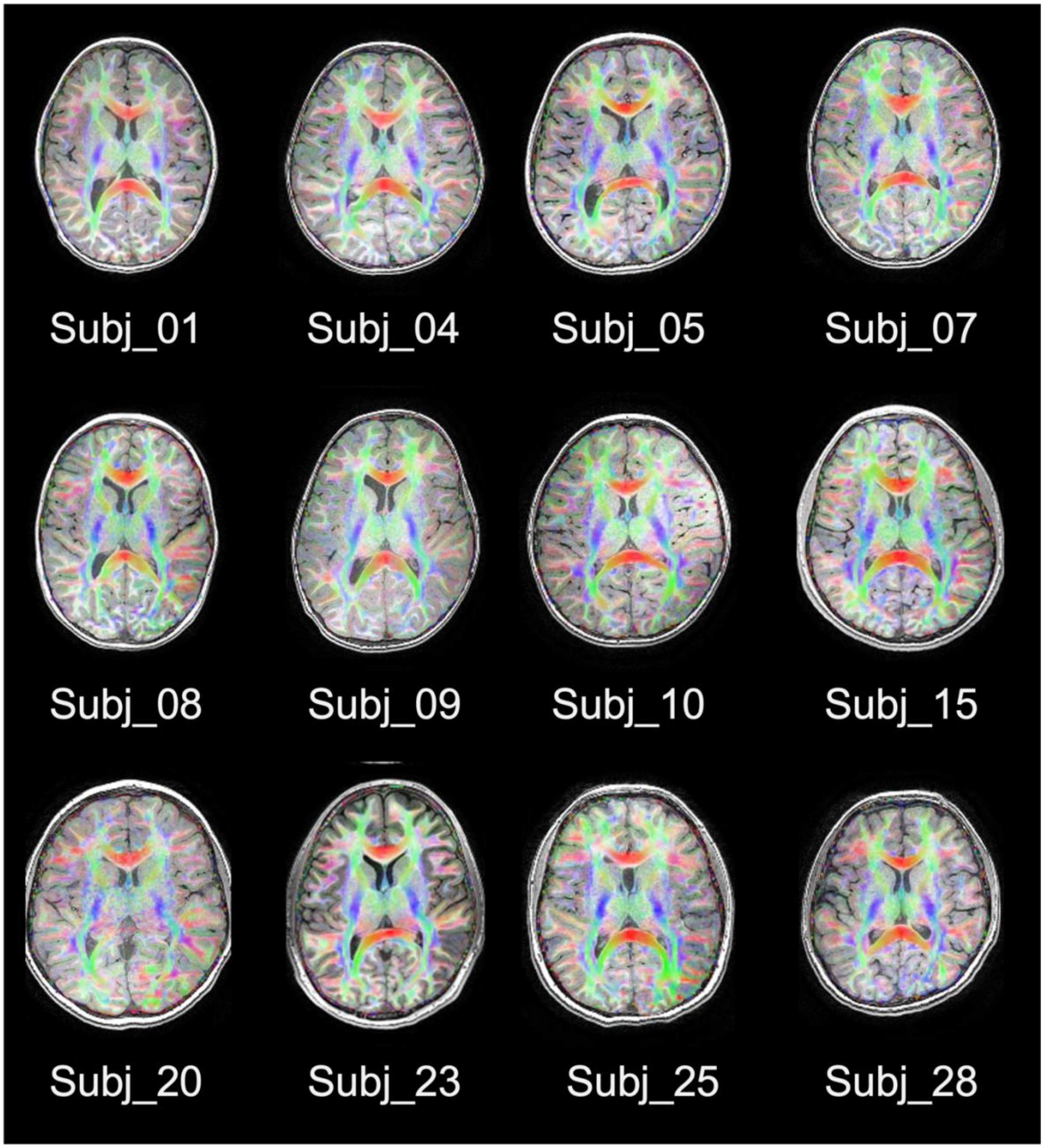


**Supplementary Figure 6.** Representative red-green-blue (RGB) color-coded fractional anisotropy (FA) maps from a randomly selected subset of participants. The principal diffusion directions are color-coded (red: left-right, green: anterior-posterior, blue: superior-inferior). These maps demonstrate the reliability of reconstructed fiber orientations, and the consistent anatomical alignment across subjects highlights the robustness of the preprocessing pipeline.
